# Role of Sigma Receptors in Alcohol Addiction

**DOI:** 10.3389/fphar.2019.00687

**Published:** 2019-06-14

**Authors:** Sema G. Quadir, Pietro Cottone, Valentina Sabino

**Affiliations:** Laboratory of Addictive Disorders, Departments of Pharmacology and Psychiatry, Boston University School of Medicine, Boston, MA, United States

**Keywords:** alcoholism, drinking, dependence, ethanol, consumption

## Abstract

Pharmacological treatments for alcohol use disorder (AUD) are few in number and often ineffective, despite the significant research carried out so far to better comprehend the neurochemical underpinnings of the disease. Hence, research has been directed towards the discovery of novel therapeutic targets for the treatment of AUD. In the last decade, the sigma receptor system has been proposed as a potential mediator of alcohol reward and reinforcement. Preclinical studies have shown that the motivational effects of alcohol and excessive ethanol consumption involve the recruitment of the sigma receptor system. Furthermore, sigma receptor antagonism has been shown to be sufficient to inhibit many behaviors related to AUDs. This paper will review the most current evidence in support of this receptor system as a potential target for the development of pharmacological agents for the treatment of alcohol addiction.

## Introduction

Alcohol is the most ubiquitously consumed mind-altering substance in the world, and it is considered responsible for 25% of the mortality in people aged 20–30. The lifetime prevalence of alcohol use disorder (AUD) in the United States is estimated to be 29.1% (Hasin et al., 2007; Grant et al., 2015).

AUD is a multifactorial complex disorder with multiple genes and environmental factors interacting to produce the phenotype of addiction (Koob and Le Moal, 2005). **Table 1** illustrates the 11 diagnostic criteria of AUD according to the most recent edition of the *Diagnostic and Statistical Manual of Mental Disorders, Fifth Edition* (DSM-V) (American Psychiatric Association, 2013). Notably, while tolerance and withdrawal were already among the criteria in previous editions, this edition includes craving for the first time, likely to convey the importance of behavioral over pharmacological components.

**Table 1:** Alcohol use disorder (AUD) diagnostic criteria.

Category	Criteria	Description
Impaired control	C1	Drinking more than intended
	C2	Unable to cut down or stop drinking
	C3	Spending a lot of time drinking or recovering from drinking
	C4	Wanting a drink so badly you can’t think of anything else (craving)
Social impairment	C5	Drinking interferes with home, family, job, or school
	C6	Drinking even though it causes trouble with friends or family
	C7	Giving up on important activities to drink instead
Risky use	C8	Drinking and getting into situations that increase chances of getting hurt
	C9	Continuing to drink despite becoming anxious, depressed, or experiencing memory blackout
Pharmacological indicators	C10	Needing to drink more to feel the same effect (tolerance)
	C11	Experiencing withdrawal symptoms such as restlessness, nausea, seizures, and hallucinations

The 11 criteria used to characterize AUD can be divided into four categories—impaired control, social impairment, risky use, and pharmacological indicators. Severity of AUD is defined by the number of symptoms present (2–3: mild, 4–5: moderate, 6 or more: severe) (American Psychiatric Association, 2013).

The negative impact of alcohol on health makes the discovery of novel potential therapeutic targets for AUD the subject of extensive research. One of these has been identified in Sigma receptors (Sig-Rs). Two subtypes of Sig-Rs have been identified, sigma-1 receptor (Sig-1R) and sigma-2 receptor (Sig-2R); this review will focus exclusively on the available evidence concerning Sig-1R. Indeed, since Sig-2R was only cloned in 2017 (Alon et al., 2017), very few selective ligands have been available and, therefore, most evidence in the context of alcohol and other addictions has been obtained using either non-selective Sig-1R/Sig-2R or Sig-1R selective ligands. Specifically, we will summarize research showing that manipulations of Sig-1R impact ethanol-induced changes in locomotor function, acquisition and expression of conditioned place preference (CPP), alcohol consumption and seeking behavior, and reinstatement of alcohol-seeking behavior, as well as deficits in cognitive function induced by alcohol.

### Sigma Receptors

Two subtypes of Sig-Rs have been described: Sig-1R and Sig-2R. While both are sensitive to haloperidol and 1,3-di(2-tolyl)guanidine, Sig-1Rs are more sensitive to benzomorphans such as (+)-SKF-10,047, dextromethorphan, and carbetapentane (Bouchard and Quirion, 1997). In addition, the two receptor subtypes differ in size, with Sig-1R having a larger molecular weight (Alon et al., 2017). Proposed endogenous ligands include N,N-dimethyltryptamine and neurosteroids (Fontanilla et al., 2009); interestingly, psychostimulants, such as cocaine, lidocaine, and methamphetamine, have all been shown to bind Sig-1R, although with rather weak affinity (Sharkey et al., 1988; Nguyen et al., 2005).

#### Sig-1R Structure and Localization

Sig-1R is a 25–29 kDa protein encoded by the *SIGMAR1* gene (Prasad et al., 1998). In 2016, Schmidt and colleagues solved the crystal structure of human Sig-1R and found it to be a trimer, with each protomer containing one transmembrane domain (Schmidt et al., 2016). The gene that encodes the human Sig-1R is found on band p13 of chromosome 9 and is ∼7 kbp long, coding four exons (encoding a 25.3 kDa protein) and three introns (Aydar et al., 2002; Matsumoto et al., 2003). Although Sig-1R shows no homology with any other known mammalian proteins, Sig-1R shares 30% identity and 67% similarity with a yeast sterol isomerase, consistent with the observation that sterol-producing tissues have high levels of Sig-1R mRNA (Balasuriya et al., 2012).

Sig-1Rs are found both intracellularly and on the plasma membrane (Aydar et al., 2002). Specifically, they have been shown to be extensively associated with cholesterol-enriched loci on the endoplasmic reticulum, as well as on the mitochondrion-associated endoplasmic reticulum membrane (Alonso et al., 2000; Hayashi and Su, 2001; Hayashi and Su, 2003). Upon activation, the receptors move laterally toward the periphery of the cell (Hayashi and Su, 2003). Sig-1Rs have been shown to be in cell bodies and on dendrites, but not on axon terminals or fibers table (Alonso et al., 2000).

#### Receptor signaling

Sig-1R has been shown to associate with a variety of proteins such as ankyrin B, heat shock protein 70, phospholipase C, and thus protein kinase C (PKC), protein kinase A, and glucose-related protein/immunoglobulin heavy-chain-binding protein, among others (Morin-Surun et al., 1999; Hayashi and Su, 2001; Matsumoto et al., 2003; Hayashi and Su, 2007; Kim et al., 2008). In addition, Sig-1Rs modulate voltage-gated ion channels, such as the N-type Ca^2+^ channel, Nav1.5, Kv1.2, Kv1.3, Kv1.4, and Kv1.5 (Aydar et al., 2002; Balasuriya et al., 2012; Kinoshita et al., 2012; Kourrich et al., 2013; Liu et al., 2017; Zhang et al., 2017). While Sig-1Rs are not G-protein coupled, they physically associate with certain G-protein coupled receptors, such as mu opioid and dopamine D1 and D2 receptors, modulating their activity (Kim et al., 2010; Navarro et al., 2010; Navarro et al., 2013). Additional studies determined that Sig-1R also modulates dopamine transporters (Hong et al., 2017).

Sig-1R activation has been shown to potentiate *N*-methyl-*D*-aspartate receptor (NMDAR)-induced Ca^2+^ influx, mainly through the facilitation of PKC-mediated phosphorylation of the GluN1 subunit (Roh et al., 2011). Sig-1R activation has also been linked to increased GluN2a, GluN2b, and postsynaptic density protein 95 expression, as well as to NMDAR subunit redistribution and increased NMDAR trafficking to the synapse (Pabba et al., 2014). In addition, Sig-1R activation potentiates the release of several neurotransmitters, such as acetylcholine, dopamine, and brain-derived neurotrophic factor (Ault and Werling, 1997; Matsuno et al., 1997; Fujimoto et al., 2012), all of which are perturbed in AUD (Moykkynen and Korpi, 2012; Engel and Jerlhag, 2014; Wu et al., 2014). This indicates Sig-1R as a critically important target for the study of AUD.

### Sigma Receptors and Locomotor Effects of Alcohol

At low doses, alcohol stimulates locomotor activity (Risinger and Oakes, 1996). This activation, which is thought to occur through the stimulation of the mesolimbic system, is hypothesized to be an index of its abuse liability (Phillips and Shen, 1996).

The role of Sig-1R in alcohol-induced locomotion has been studied in mice. Although Sig-1R agonists and antagonists *per se* fail to influence locomotor activity, Maurice and colleagues demonstrated that Sig-1R blockade* via* the selective antagonist N-[2-(3,4-dichlorophenyl)ethyl]-N-methyl-2-(dimethylamino)ethylamine (BD-1047) inhibits the increase in locomotor activity induced by 1 g/kg of ethanol, in a dose-dependent manner (Maurice et al., 2003). However, treatment with the Sig-1R selective agonist 2-(4-morpholine) ethyl 1-phenylcyclohexame-1-carboxylate (PRE-084) fails to influence alcohol-induced locomotion (Maurice, 2003). This could be indicative of Sig-1R antagonists decreasing the response of the mesolimbic dopamine system. Interestingly, Sig-1R agonists have been shown to increase cocaine and methamphetamine-induced locomotor activity, while antagonists have been shown to decrease this response (Takahashi et al., 2000; Liu and Matsumoto, 2008). The reason why Sig-1R agonists increase cocaine and methamphetamine-induced locomotor activity but fail to influence alcohol-induced locomotor activity is unclear, and it may be related to the specific mechanisms of action of psychostimulants.

Despite the fact that ethanol-induced locomotion stimulation is easily observed in Swiss mice, C57BL/6J mice are not as sensitive to the stimulatory effects of ethanol (Becker and Hale, 1989; Maurice et al., 2003; Valenza et al., 2016). To unmask the sedative effects of alcohol in C57BL/6J mice, ethanol is often co-administered with the benzodiazepine partial inverse agonist RO15-4513 (Becker and Hale, 1989). Valenza and colleagues (2016) employed this technique to examine the stimulatory effects in C57BL/6J lacking the *SIGMAR1* gene, which encodes the Sig-1R. Sig-1R KO mice were found to be less sensitive to the locomotor stimulating effects of 1.5 g/kg alcohol when compared to WT (Valenza et al., 2016). As the stimulant effects of alcohol are thought to be related to its motivational and rewarding properties (Phillips and Shen, 1996), the reduced sensitivity of Sig-1R KO mice to the stimulant effects of alcohol may reflect a reduced sensitivity to its motivational effects, which may, in turn, be responsible for their increased alcohol intake (i.e., higher amounts of alcohol required to feel euphoric effects) (Valenza et al., 2016).

At high doses, alcohol acts as a central nervous system depressant. A single study investigated the effects of Sig-1R ligands on ethanol-induced sedation and found that Sig-1R KO do not differ from WT in either loss of righting reflex or sleep duration, suggesting that Sig-1R is not involved in the sedative effects of ethanol (Valenza et al., 2016). These studies corroborate the notion that Sig-1Rs are involved in the stimulatory, but not the sedating, effects of ethanol.

### Sigma Receptors and the Rewarding Properties of Alcohol

One of the most common tests to examine the rewarding properties of substances in animals is the place conditioning paradigm. The systemic administration of the selective Sig-1R agonist PRE-084 prior to ethanol enhances ethanol-induced CPP (Maurice et al., 2003). Studies using intracerebroventricular administration of the same agonist also showed a facilitation of the acquisition of ethanol-induced CPP (Bhutada et al., 2012). Moreover, pretreatment with the Sig-1R antagonist BD-1047, administered during conditioning, blocks the acquisition of ethanol-induced CPP (Maurice et al., 2003), while its intracerebroventricular administration has been shown to reduce both acquisition and expression (Bhutada et al., 2012). Lastly, neither Sig-1R agonists nor antagonists affect place preference when administered alone (Romieu et al., 2000; Maurice et al., 2003). Altogether, these studies show that Sig-Rs bidirectionally modulate the rewarding properties of alcohol.

### Sigma Receptors and Alcohol Drinking

Evidence from both human and animal studies strongly implicates Sig-1Rs involvement in alcohol drinking. In relation to preclinical studies, these have all been performed in rodents and they can be divided into two types: passive home cage alcohol drinking studies and active operant alcohol self-administration studies.

#### Home Cage Alcohol Drinking

Many home cage drinking studies have been performed in The Scripps Research Institute (TSRI) Sardinian alcohol-preferring rats (Scr:sP), a line that descends directly from Sardinian alcohol preferring rats, which were selectively bred to drink high amounts of alcohol (Colombo et al., 2006; Sabino et al., 2013). Ethanol-naïve Scr:sP rats have elevated Sig-1R levels in the NAcc when compared to ethanol-naïve outbred Wistar rats (Blasio et al., 2015). After 4 weeks of voluntary ethanol consumption, Sig-1R levels in the NAcc of Scr:sP rats returned back to baseline, suggesting that this could be a mechanism for the reduced motivation to drink after chronic drinking (Blasio et al., 2015). Interestingly, the changes in protein level only occurred in the NAcc and not in the central nucleus of the amygdala, perhaps implicating the mesolimbic dopaminergic system (Blasio et al., 2015). This is consistent with previous studies demonstrating the modulation of dopaminergic neurotransmission by Sig-1Rs (Ault and Werling, 1999; Navarro et al., 2010; Navarro et al., 2013). In line with these findings, the selective Sig-1R antagonists NE-100 and BD-1063 both reduce ethanol home cage drinking in Scr:sP rats, without affecting total fluid intake or food intake (Sabino et al., 2009b; Blasio et al., 2015). NE-100 also fails to affect food intake or sucrose preference, suggesting that the effects of the drug are selective for alcohol (Sabino et al., 2009b). However, in that study, rats developed tolerance to NE-100 treatment within 1 week, similar to opioid receptor antagonist treatments (Overstreet et al., 1999; Sabino et al., 2009b).

Sig-1R KO mice exhibit increased alcohol preference and drink more alcohol compared to WT mice (Sabino et al., 2009b; Valenza et al., 2016). The effect observed is specific for alcohol, as genotypes did not differ in their intake or preference for either the bitter tasting quinine or the sweet tasting saccharin, ruling out altered taste perception (Valenza et al., 2016). The data obtained in Sig-1R KO may appear counterintuitive and in contrast with the general hypothesis that Sig-1R hyperactivity contributes to excessive ethanol intake. However, it is possible that developmental compensatory changes in expression of other genes may occur in Sig-1R KO mice, e.g., an up-regulation of Sig-2Rs, which may explain some of the observed effects.

#### Operant Alcohol Self-Administration

A method used to assess the reinforcing properties of alcohol is the use of operant conditioning, where rodents must lever press in order to obtain alcohol. Sig-R activation *via* daily systemic treatment of the Sig-1R/Sig-2R agonist 1,3-di-(2-tolyl)guanidine (DTG) is able to induce binge-like drinking (defined as blood alcohol concentration >80 mg/dl) in Scr:sP rats under a fixed ratio 1 ratio of reinforcement, effect blocked by the selective Sig-1R antagonist BD-1063 (Sabino et al., 2011). Consistent with a bidirectional modulation of alcohol self-administration, treatment with the selective Sig-1R antagonist BD-1063 decreases ethanol responding in Scr:sP rats in a dose-dependent manner (Sabino et al., 2009a).

Another schedule of reinforcement widely used in psychopharmacological research on alcohol is the progressive ratio schedule of reinforcement, which allows studying subjects’ motivation to obtain the reinforcer (Hodos, 1961). Daily systemic treatment of DTG increases, whereas acute BD-1063 administration decreases, the breakpoint for alcohol in Scr:sP rats, thus demonstrating the bi-directional role of Sig-1R also in modulating the motivational properties of ethanol (Sabino et al., 2009a; Sabino et al., 2011).

Additional studies examined the role of Sig-1R in alcohol dependence in Wistar rats. Dependence was induced *via* chronic intermittent ethanol (CIE) vapor exposure, and this method has been shown to result in high blood alcohol levels (150–200 mg%), compulsive drinking, elevated anxiety, and increased ethanol intake during withdrawal (for review, see Vendruscolo and Roberts, 2014). Pretreatment with the selective Sig-1R antagonist BD-1063 reduces ethanol self-administration in dependent, but not in non-dependent, outbred Wistar rats, without affecting water or saccharin intake, suggesting that Sig-1Rs modulate both genetic and environmental excessive drinking (Sabino et al., 2009a). In addition, compared to ethanol naïve Wistar rats, ethanol-dependent Wistar rats during acute withdrawal and ethanol naïve Scr:sP rats had significantly less Sig-1R mRNA in the NAcc, indicating that repeated cycles of intoxication followed by withdrawal mirror the phenotype observed in Scr:sP rats (Sabino et al., 2009a).

Lastly, repeated DTG administration in Scr:sP rats results in an increase in μ- and δ-opioid receptor gene expression in the ventral tegmental area (VTA) (Sabino et al., 2011), indicating that Sig-R activation may lead to disinhibition of the mesolimbic dopaminergic VTA-NAcc pathway, resulting in increased dopamine release and increased reward sensitivity.

### Sigma Receptors and Relapse-Like Behaviors

Relapse after abstinence remains one of the most problematic barriers to treating AUDs. Cravings, or tenacious urges, to drink alcohol or engage in alcohol-seeking behaviors often occur during periods of abstinence and result in relapse (Martin-Fardon and Weiss, 2013). In rodents, this may be modeled by exposing animals to alcohol, then forcing them to abstain for a period of time, and lastly presenting alcohol again. Under these conditions, vehicle-treated abstinent Scr:sP rats dramatically increase their ethanol intake upon renewed presentation of alcohol, and blocking Sig-1R with the selective Sig-1R antagonist NE-100 blocks this ethanol deprivation effect (Sabino et al., 2009b). This study thus paves the way for more research into the role of Sig-1R in behaviors related to alcohol-seeking and relapse.

Another procedure is the priming-induced alcohol-seeking method, which mimics the human condition known as “one drink, one drunk,” initially described by Jellinek and later validated by Hodgson and colleagues (Jellinek, 1952; Hodgson et al., 1979). In the context of reinstatement of ethanol-induced CPP, the administration of the selective Sig-1R agonist PRE-084 intracerebroventricularly was shown to be sufficient to induce reinstatement (Bhutada et al., 2012). Furthermore, pretreatment with the Sig-1R antagonist BD-1047 dose-dependently blocks both ethanol-induced and PRE-084-induced reinstatement, thus confirming that Sig-1R activation is required for priming-induced reinstatement (Bhutada et al., 2012).

Another important factor for relapse is the powerful effect that drug-paired cues exert on behavior. Selectively antagonizing Sig-1R with BD-1047 blocks reinstatement induced by ethanol- or palatable food-associated cues (Martin-Fardon et al., 2012), thus indicating that this modulation is not specific to drug reward as it also applies to seeking behavior for highly palatable food. In a seeking-taking chained second-order schedule of reinforcement, in which an alcohol-associated incentive stimulus maintains alcohol-seeking behavior (Everitt, 2014), the Sig-1R selective antagonist BD-1063 dose-dependently reduces alcohol-seeking behavior (Blasio et al., 2015), suggesting a role for Sig-1R in incentive motivational mechanisms controlling ethanol-seeking and intake. Together, these studies provide insight into the critical role of the Sig-1R system in relapse-related behaviors.

### Sigma Receptors and Cognitive Impairment During Alcohol Withdrawal

Withdrawal after chronic alcohol consumption has been linked to deficits in cognitive function in human subjects (Sabia et al., 2011). While the Sig-R system has been highly implicated in cognitive function (Matsuno et al., 1997), only few studies have examined the putative role of Sig-R system in chronic alcohol-induced cognitive impairment.

Using the novel object recognition task, Meunier and colleagues (2006) assessed cognitive function during protracted withdrawal in mice that had undergone chronic alcohol consumption (4 months). Alcohol withdrawn mice showed increased anxiety-like behaviors, elevated locomotion, and impaired object recognition; systemic administration of either the Sig-1R selective antagonist BD-1047 or the selective agonist igmesine is able to restore the habituation response (defined as decreased interactions with previously presented objects), but only the latter corrected reactions to spatial change and novelty (Meunier et al., 2006). These mice also show upregulated hippocampal Sig-1R expression, which normalized after repeated administration of either Sig-1R ligand (Meunier et al., 2006), indicating that Sig-1R levels may be responsible for the cognitive deficits seen in alcohol withdrawal.

Although the object recognition task provides direct evidence for the role of Sig-1R in cognition, other studies have used slice electrophysiology to examine the role of Sig-1Rs in hippocampal long-term potentiation (LTP). Initial studies that investigated the effect of CIE *via* vapor on LTP development in stimulatory CA1 synapses found juvenile rats in withdrawal from CIE vapors and increased NMDAR independence compared to their non-CIE vapor-treated counterparts (Sabeti and Gruol, 2008). Furthermore, this increase in NMDAR independence in LTP is blocked by administration of the Sig-1R antagonist BD-1047, thus providing evidence for the role of the Sig-1R.

Subsequent studies found that early adolescent CIE vapor rats in withdrawal show depressed LTP excitability after high amplitude stimulus compared to controls (Sabeti, 2011). The decreases in action potential spike amplitude and excitatory postsynaptic potentiation in these subjects are reverted back to normal values by the Sig-1R antagonist BD-1047, suggesting that alcohol withdrawal may activate Sig-1Rs and therefore dampen the excitatory inputs during LTP (Sabeti, 2011). A recent study found that the decreased levels of neuron-specific nuclear protein/Fox-03 caused by CIE application to hippocampal explants can be reversed by treatment with the Sig-1R antagonist BD-1047 (Reynolds et al., 2016).

Together, these results indicate a strong role for Sig-1Rs in alcohol withdrawal-related neuroadaptations and symptomatology.

### Limitations

The studies reviewed in this manuscript have all focused on preclinical work performed in rodents. However, it is important to note that many of the studies involve animals consuming high amounts of alcohol and, therefore, caution should be exerted before extrapolating the results to humans. A single human study has so far shown a possible role for Sig-1R in AUD, specifically that a Japanese population of alcoholic subjects display three polymorphisms in the 5’ untranslated region of the gene coding Sig-1Rs (*SIGMAR1*) that are highly associated with alcoholism (Miyatake et al., 2004). Additional human studies are indeed warranted to confirm that the rodent studies mentioned in this review have a translational value.

It is also important to note that all of the studies described in this review were performed in male animals. Since some neurobiological mechanisms of AUD are sexually dimorphic and alcohol use does differ between men and women (Erol and Karpyak, 2015), further studies hold potential for providing essential information regarding this gap.

### Concluding Remarks

The Sig-R system is proving to be a promising pharmacological target for AUD treatment. In various animal models, Sig-1R antagonists reduce alcohol consumption, motivation to drink, and alcohol-seeking behavior, demonstrating a critical role for Sig-1Rs in these behaviors (see **Table 2** **)**. Since current Food and Drug Administration (FDA)-approved medications for AUD, i.e., disulfiram, naltrexone, and acamprosate, have been shown to have limited efficacy, Sig-1R targeting drugs could represent a promising therapeutic option. Yet, the precise routes of action through which the Sig-1R system impacts alcohol’s effects are unknown, and therefore, additional studies to unveil these mechanisms and the relationship between Sig-1R and alcohol will be vital to comprehending AUD from a neurobiological standpoint and developing novel and more efficacious pharmacological therapeutics.

**Table 2:** Summary of the pharmacological findings included in this review.

Behavior	Drug type	Results	Citation
Locomotor	Antagonist (BD-1047)	Inhibited ethanol-induced (1 g/kg) locomotor activity	(Maurice et al., 2003)
	Agonist (PRE-084)	No effect on ethanol-induced (0.5 g/kg) locomotor activity	(Maurice et al., 2003)
	Genetic KO	Inhibited ethanol-induced (1.5 g/kg) locomotor activity	(Valenza et al., 2016)
	Genetic KO	No differences in sedative effects of alcohol	(Valenza et al., 2016)
Rewarding properties	Agonist (PRE-084)	Enhanced CPP	(Bhutada et al., 2012; Maurice et al., 2003)
	Antagonist (BD-1047)	Diminished CPP	(Bhutada et al., 2012)
Home cage drinking	Antagonist (NE-100)	Reduced ethanol intake	(Sabino et al., 2009b)
	Antagonist (BD-1063)	Reduced ethanol intake	(Blasio et al., 2015)
	Genetic KO	Increased ethanol intake and preference	(Valenza et al., 2016)
Operant self-administration	Agonist (DTG)	Induce binge-like drinking	(Sabino et al., 2011)
	Antagonist (BD-1063)	Reduced ethanol intake	(Sabino et al., 2009a)
Motivation to drink	Agonist (DTG)	Increased breakpoint	(Sabino et al., 2011)
	Antagonist (BD-1063)	Decreased breakpoint	(Sabino et al., 2009a)
Alcohol deprivation effect	Antagonist (NE-100)	Inhibited alcohol deprivation effect	(Sabino et al., 2009b)
Reinstatement of CPP	Agonist (PRE-084)	Induced CPP	(Bhutada et al., 2012)
	Antagonist (BD-1047)	Inhibited ethanol and PRE-084-induced reinstatement	(Bhutada et al., 2012)
Reinstatement of operant behavior	Antagonist (BD-1047)	Inhibited reinstatement of both food and alcohol cue-induced reinstatement	(Martin-Fardon et al., 2012)
Seeking-taking chained schedule of reinforcement	Antagonist (BD-1063)	Reduced alcohol-seeking	(Blasio et al., 2015)
Cognitive impairment during alcohol withdrawal	Agonist (igmesine)	Restored cognitive responses in withdrawn mice	(Meunier et al., 2006)
	Antagonist (BD-1047)	Restored cognitive responses in withdrawn mice	(Meunier et al., 2006)
LTP	Antagonist (BD-1047)	Blocks increase in NMDAR-independent LTP seen in withdrawn rats	(Sabeti and Gruol, 2008)
	Antagonist (BD-1047)	Blocks action potential spike amplitude and excitatory post-synaptic potentiation in withdrawn rats	(Sabeti, 2011)

This table summarizes the major findings presented.

BD-1047, N-[2-(3,4-dichlorophenyl)ethyl]-N-methyl-2-(dimethylamino)ethylamine; PRE-084, 2-(4-morpholine) ethyl 1-phenylcyclohexame-1-carboxylate; KO, sigma-1 knockout; NMDAR, N-methyl-D-aspartate receptor; LTP, long-term potentiation; BD-1063, 1-[2-(3,4-dichlorophenyl)ethyl]-4-methylpiperazine; NE-100, 4-methoxy-3-(2-phenylethoxy)-N,N-dipropylbenzeneethanamine; CPP, conditioned place preference; DTG, 1,3-Di-o-tolylguanidine.

## Author Contributions

All authors made substantial contributions to conception and design of this review. SQ drafted the manuscript. VS and PC substantially and critically revised it for intellectual content. All authors gave final approval for its submission.

## Funding

This work was supported by the National Institutes on Alcohol Abuse and Alcoholism [grant numbers AA024439 (VS), AA025038 (VS), and AA026051 (PC)] and the Burroughs Wellcome Fund (SQ) through the Transformative Training Program in Addiction Sciences. Its contents are solely the responsibility of the authors and do not necessarily represent the official views of the National Institutes of Health.

## Conflict of Interest Statement

The authors declare that the research was conducted in the absence of any commercial or financial relationships that could be construed as a potential conflict of interest.
